# Identification and Phylogenetic Analysis of *Mycobacterium avium* subsp. *avium* Strain Isolated from Cow

**DOI:** 10.1155/2023/5384079

**Published:** 2023-06-01

**Authors:** Yanhong Bao, Tianze Yang, Hanxue Zhang, Zi Wang, Inam Muhammad, Xiuyun Jiang, Hongxia Ma

**Affiliations:** ^1^College of Life Sciences, Jilin Agricultural University, Xincheng Street No. 2888, Changchun 130118, China; ^2^College of Animal Science and Technology, Inner Mongolia University for Nationalities, Tongliao 028000, China; ^3^Department of Animal Sciences, Shaheed Benazir Bhutto University Sheringal, Dir Upper, Pakistan; ^4^College of Animal Medicine, Jilin Agricultural University, Xincheng Street No. 2888, Changchun 130118, China; ^5^College of Life Sciences, Changchun Sci-Tech University, Changchun 130600, China; ^6^The Key Laboratory of New Veterinary Drug Research and Development of Jilin Province, Jilin Agricultural University, Xincheng Street No. 2888, Changchun 130118, China; ^7^The Engineering Research Center of Bioreactor and Drug Development, Ministry of Education, Jilin Agricultural University, Xincheng Street No. 2888, Changchun 130118, China

## Abstract

*Mycobacterium avium* subsp. *avium* (MAA) is the main tuberculosis pathogen of poultry and wild birds. MAA can also infect mammals such as pigs, cattle, and horses and can pose a threat to people with low immunity. Here, we describe the first identification of MAA strain HJW isolated from a cow in Jilin Province, China. The isolate was completely sequenced and a phylogenetic analysis of its relationship to members of the *Mycobacterium avium* complex (MAC) was performed. The results revealed that strain HJW was type MAA based on the analysis of insertion sequence amplification and whole genome sequencing. The HJW genome size was 4,961,843 bp with a GC content of 69.28%. The strain was genetically most closely related to the *Mycobacterium avium* subsp. *avium* strain DSM 44156. This study suggests that MAA may pose an infection risk to cattle and provides data support for the phylogeny of *Mycobacterium avium*.

## 1. Introduction

Currently, cases of diseases caused by nontuberculous mycobacteria (NTM) are rapidly increasing. NTM are known to be ubiquitous in the environment and capable of widespread transmission in animals and humans [1–6]. *Mycobacterium avium* and *Mycobacterium intracellulare* are the two main species belonging to the *Mycobacterium avium* complex (MAC). *Mycobacterium avium* is one of the most common pathogenic bacteria causing human and animal diseases worldwide and is an important opportunistic pathogen [7, 8]. The respiratory tract, lymph nodes, and some soft tissues are common sites of infection. Moreover, secondary infections with *Mycobacterium avium* have gained more attention due to the recent increase in immunocompromised individuals, such as AIDS patients [9, 10]. *Mycobacterium avium* has four sub-subspecies: *M. avium* subsp. *avium* (MAA), *M. avium* subsp. *silvaticum* (MAS), *M. avium* subsp. *paratuberculosis* (MAP), and *M. avium* subsp. *hominissuis* (MAH) [11, 12].

MAA is considered to be a major avian pathogen and is the causative agent of avian mycobacteriosis. According to World Organization for Animal Health, avian mycobacteriosis is defined as type B disease [13]. MAA not only has economic significance to various animals and endangers animal health [14–18] but also occasionally causes infections and diseases in humans [19] leading to potential zoonotic hazards. At present, although a great deal of attention has been paid to MAC research in other parts of the world, but there have been only limited reports of the isolation of MAC strains in China [20–24], and only one case of a MAA strain related outbreak has been reported in recent years [25].

Birds, especially poultry, are highly susceptible to MAA and are usually the first to contract the disease and become the main reservoir of bacteria. Infected farmed birds can spread MAA to susceptible animals via feces, wild birds, rodents, or through fecal polluted environments [26]. Avian mycobacteriosis usually has many clinical manifestations such as depression, weight loss, and decreased egg production [27]. Tuberculosis nodules are common in the spleen, intestines, liver, and bone marrow and less frequently in other organs. The typical symptom is calcified granulomatous lesions [28]. In addition to birds and common domestic poultry, MAA can infect mammals such as cattle, pigs, and horses [29–32] and may even pose a threat to humans [33]. However, different species have different chances of contracting diseases. Besides birds and pigs are susceptible to infection caused by MAA. *M. avium* serovars 1–6 and 8–11 have been isolated from swine [34]. MAA has frequently been isolated from tuberculosis (TB)-like lesions found in slaughtered pigs [35–37]. In pigs, the disease caused by isolates of MAA often occurs as lymphadenitis and rarely as parenchymatous tuberculosis [38]. In addition, in swine, mycobacterial infection usually occurs without clinical manifestation. Unlike MAP, MAA is relatively nonpathogenic for cattle. Organisms appear to enter through the intestinal tract and localize in lymph nodes. Cattle typically mount an effective systemic immune response, form caseous granulomas, and eliminate the organisms [39].

In addition, MAA has a complex thick cell wall that impedes the permeation of multiple compounds and is responsible for the intrinsic multidrug resistance and virulence of these bacteria. This has mainly been attributed to the unusually thick and lipid-rich cell envelope [40]. After penetrating the cell envelope, certain antibiotics are cleaved enzymatically or altered structurally to render them ineffective. Apart from the intrinsic resistance mechanisms mentioned above, the majority of clinically relevant cases of drug resistance in *Mycobacterium tuberculosis* are conferred by chromosomal mutations. These chromosomal mutations confer drug resistance via a large array of different mechanisms and may confer different levels of resistance [41]. Furthermore, the existence of specific antibiotic resistance genes and mutations in some genes (such as rpsL and rpoB genes) that have adverse effects on antimicrobial activities may be several factors conferring MAA multidrug resistance [19]. Recently, Viale et al. used the indirect proportion method to measure drug resistance in MAA. It was shown to be resistant to isoniazid (INH), rifampicin (RIF), levofloxacin (LX), and p-nitro-benzoic acid (PNB) [42].

Therefore, accurate identification of *Mycobacterium avium* subspecies is critical to better understand the underlying mechanisms of disease and to develop more beneficial control plans. Genetic analysis assay is a valuable alternative to previous typing methods for defining the MAC subspecies. Due to the high sequence similarity between MAA and MAS, they are usually indistinguishable through phenotype and classical molecular methods. In the present study, we have reported the identification of a MAA strain isolated from a cow and have described the genomic and phylogenetic characterization of the isolate.

## 2. Methods

### 2.1. Bacterial Isolation

A Holstein–Friesian cow presented at a dairy farm with diarrhea and weight loss and unsuccessful antimicrobial and cortisone therapy. A fecal sample was collected from the rectum and *Mycobacterium* cells in phosphate buffered saline (PBS) were obtained by methods reported in previous studies [26]. The mixture was inoculated onto Löwenstein–Jensen solid medium (Hopebiol, Co., Ltd., Qingdao, China) for 6 weeks to obtain individual bacterial colonies. The bacteria were subcultured with 7H9 broth (BD) supplemented with OADC (BD) and incubated at 37°C.

### 2.2. Staining Cell Colonies and DNA Preparation

The grown colonies on the solid medium were checked by Gram staining and Ziehl–Neelsen acid-fast staining. Colonies were screened to identify those containing Gram-positive bacteria and acid-fast bacteria (AFB) for further analysis. DNA for PCR testing was extracted from bacteria grown in 7H9 broth. To confirm the PCR results, a *Mycobacterium avium* subsp. *paratuberculosis* strain (MAP-10) was used.

### 2.3. Polymerase Chain Reaction (PCR)

The five target genes 16S rRNA, IS1311, DT1, IS900, and IS901 were amplified by PCR to achieve further identification [43]. All primers are as reported in previous literature [43]. PCR reaction conditions were 95°C predenaturation, 5 min; denaturation at 95°C for 1 min; annealing at 60°C for 1 min; and extension at 72°C for 1 min. After 30 cycles, the abovementioned three steps were extended again for 10 min at 72°C. After the reaction, PCR products were detected by 1% agarose gel electrophoresis. The amplifications of 16S rRNA, IS1311, and IS900 were interpreted as MAP-10, whereas amplification of target genes 16S rRNA, DT1, IS1311, and IS901 were interpreted and as MAA or MAS.

### 2.4. Whole Genome Sequencing and Phylogenetic Analysis

For whole genome sequencing genomic DNA was extracted with the SDS method [44]. The harvested DNA was detected by the agarose gel electrophoresis and quantified by Qubit2.0 Fluorometer (ThermoFisher Scientific, Inc., USA). Whole genome sequencing was performed on the Illumina HiSeq 2500-PE125 platform with MPS (massively parallel sequencing) Illumina technology [45]. A-tailed, ligated to paired-end adaptors and PCR amplified with a 500 bp insert and a mate-pair library with an insert size of 5 kb were used for the library construction.

Since a proportion the original data obtained by sequencing was of poor quality, it was necessary to filter the raw data to obtain high quality data for further analysis. The specific processing steps are as follows: (1) removing reads containing low-mass bases (mass value ≤ 38) exceeding a certain proportion (the default is 40 bp); (2) reads with N-bases up to a certain proportion (the default is 10 bp) were removed; (3) removing reads whose overlap with the adaptor exceeds a certain threshold (the default is 15 bp); (4) for small genomes and other projects, if the samples are contaminated by the host, they should be compared with the host database to filter out the reads that may come from the host.

Filtered data were assembled using SMRT portal assembly software [46]. Firstly, different K-mer values were selected for assembly, and then the preliminary assembly results were obtained by using the optimal kmer and adjusting other parameters (-d-u-R-F, etc.). Final assembly results were obtained after employing Krskgf, gapclose, and other software optimize and fill holes in the initial assembly results. Fragments below 500 bp were filtered out, and the remaining fragments were evaluated, statistically analyzed, and subsequent gene prediction was carried out.

The composition of the sample genome was analyzed from the completed assembly, including determination of the predicted coding genes, noncoding RNA, repetitive sequences, and gene islands. Functional annotation was performed with several different databases for coding gene sequences, including commonly used Gene Ontology (GO), Kyoto Encyclopedia of Genes and Genomes (KEGG), Cluster of Orthologous Groups of proteins (COG), and databases for pathogenic microorganisms. Finally, combining all the above analysis contents, a more basic and comprehensive analysis and display of the genome was carried out as described in [47].

### 2.5. Comparative Genome Analysis

#### 2.5.1. Phylogenetic Analysis

Phylogenetic analysis was performed using the maximum likelihood method implemented in PhyML [48], with a 1000 bootstrap replicates. The complete genomes of 9 *Mycobacterium avium* complex (MAC) strains were referenced from GenBank.

#### 2.5.2. Distribution Analysis of Virulence Factors

In order to understand the differences in the clusters of virulence factors among different subspecies of *Mycobacterium avium*, the blastall software [49] was used to compare and analyze of main virulence factors in mycobacteria.

## 3. Results

### 3.1. Bacterial Isolation and Identification

The bacteria showed typical growth of *Mycobacterium* spp on the solid medium after 4 weeks. The liquid subculture became positive after 2 weeks of incubation. The grown colonies were stained and blue-purple were declared as Gram positive, which contained straight or curved rod-shaped bacteria. While Ziehl–Neelsen acid-fast staining declared positive with red staining. The abovementioned results indicated that the colonies were AFB, i.e., mycobacteria.

### 3.2. Genetic Characterization of the Isolate

The extracted genomic DNA of *Mycobacterium avium* HJW and MAP-10 strains were used as templates to amplify 16S rRNA gene, IS1311, IS900, IS901, and DT1 genes, respectively. The 484 bp and 608 bp products from the 16S rRNA gene and IS1311 found in HJW and MAP-10 (Supplementary Figures 1A and 1B). As shown in Supplementary Figures 1C and 1D, 753 bp and 296 bp bands corresponding to IS901 and DT1 genes were amplified in HJW strain, which indicated that the strain was MAA or MAS subspecies. The MAP-10 strain was amplified to obtain 398 bp bands in accordance with IS900 genes, which proved that the strain was a subspecies of MAP-10 as shown in Supplementary Figure 1E.

AFB isolate was interpreted as MAA or MAS by the amplification results of the target genes 16S rRNA, DT1, IS*1311*, and IS*901*; therefore, it was necessary to distinguish them through further genome sequencing and phylogenetic analysis.

### 3.3. Results of HJW Strain Whole Genome Sequencing

We performed whole genome sequencing to determine the subspecies of the isolated HJW strain. After filtering the original data, the number of effective reads is 112926, the average reading length is 12264 bp, and the average sequencing quality score is 0.86. Using SMRT portal software to assemble reads, the preliminary assembly results which can reflect the basic situation of sample genome were obtained. Results of three contigs with a total length of 5,031,542 bp were obtained.

**Figure 1:**
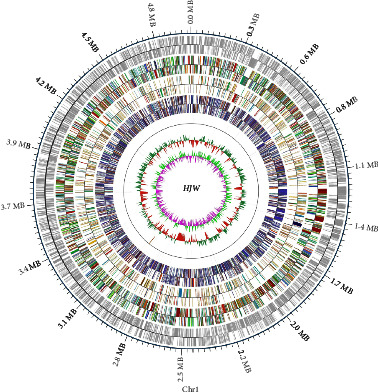
HJW Whole Genome Atlas. The outermost circle is the position coordinates of the genome sequence. From the outside to the inside, they are the coding gene, gene function annotation result, ncRNA, genome GC content, and genome GC skew value distribution.

The reads were aligned to the assembled genome sequence, and the distribution of sequencing depth of the longest sequence was statistically mapped. The preliminary assembly results were compared and analyzed, and the chromosome and plasmid sequences were screened, and the chromosome sequences were assembled into a circular genome (if it is a linear genome, it is a linear genome sequence), and the final 0gap HJW strain completion sequence was obtained as shown in Figure 1. The full length of HJW genome is 4961843 bp, with a GC content of 69.28%, 4658 predicted CDS, 47 tRNA genes, and a single rRNA operon with 16S, 23S, and 5S rRNA genes. The HJW genome sequencing results have been uploaded to the Genebank of NCBI, accession number: CP028731.

### 3.4. Results of HJW Genome-Wide Bioinformatics Analysis

GeneMarkS software [50] was used to predict the coding genes of the HJW strain genome. RepeatMasker software [51] was used to sequence the scattered repeats of the genome, and TRF (Tandem repeats finder) searches for tandem repeats in the DNA sequence. There were 16 long terminal repeats, 5 DNA transposons, 2 long scattered repeats, 7 short scattered repeats, and no rolling loop and unknown sequences in the HJW genome. See Supplementary Table 1 for details. In addition, there were 582 tandem repeats, 548 small satellite DNAs, and 2 microsatellite DNAs in the HJW genome, as shown in Supplementary Table 2.

A total of 47 tRNAs were predicted by tRNAscan-SE software. The rRNA was predicted by rRNAmmer software, and 5S rRNA, 16S rRNA, and 23S rRNA were predicted, in single copy. The Rfam database was used to compare and annotate the sRNA, and a total of 8 sRNAs were predicted.

Gene islands can be related to various biological functions such as pathogenic mechanism and adaptability of organisms. The specificity and source of functions of microorganisms with special functions can be studied by means of comparative genomic analysis. IslandPath-DIOMB software [52] was used to predict genomic islands in the HJW genome and revealed a total of 13 gene predicted islands. The specific location information for these is shown in Supplementary Table 3.

### 3.5. GO, KEGG, and COG Enrichment Analyses of HJW Gene

**Figure 2:**
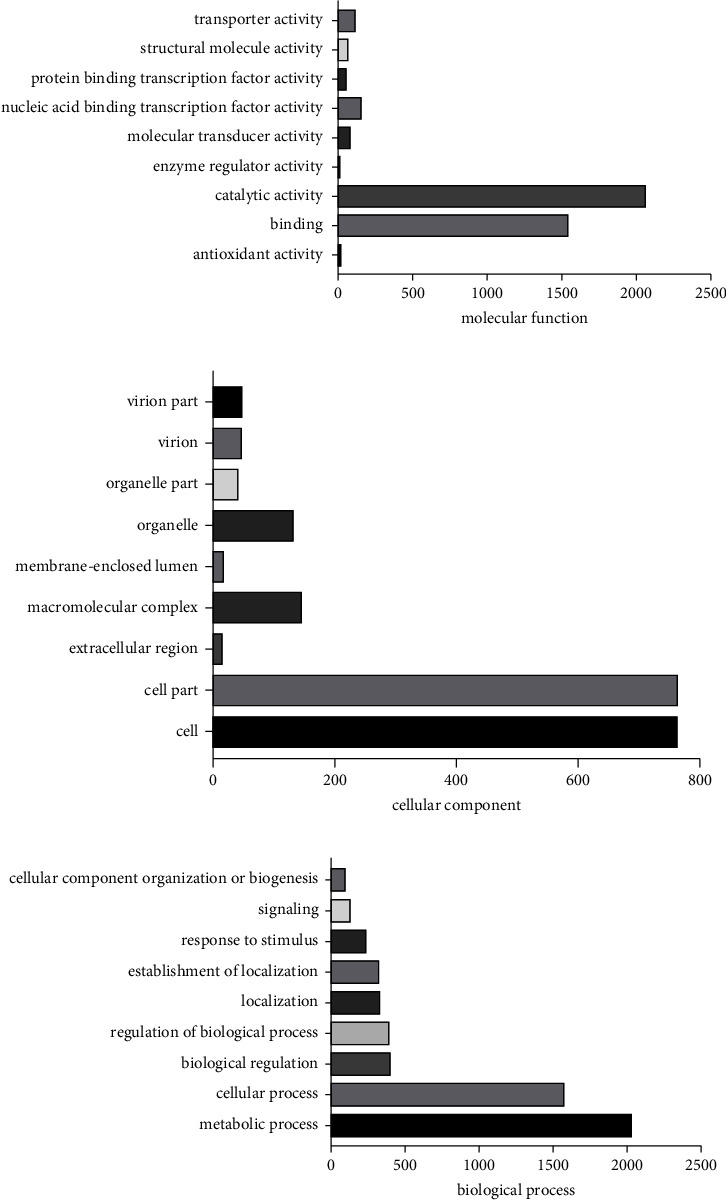
(a) Histogram of GO enrichment of HJW. The first 9 GO enrichment maps of molecular function. The abscissa is the number of genes, and the ordinate is the GO function classification. (b) Histogram of GO enrichment of HJW. The first 9 GO enrichment maps of cellular component. The abscissa is the number of genes, and the ordinate is the GO function classification. (c) Histogram of GO enrichment of HJW. The first 9 GO enrichment maps of biological process. The abscissa is the number of genes, and the ordinate is the GO function classification.

In order to analyze the function and role of predicted HJW genes, we carried out GO, KEGG, and COG enrichment analyses. GO analysis of HJW genes showed that they were involved in biological processes, cell composition, and molecular function (Supplementary Table 4). In the molecular function classification, the most significant group was the nuclear acid binding transcription factor activity for which there were 150 predicted and annotated genes, followed by catalytic activity, with 109 annotated genes identified. In the cellular component classification group, a total of 760 annotated genes were enriched in cell and cell part, respectively. In the classification of biological processes, the most important was the metabolic process, with a total of 2019 annotated genes. The second was the cellular process, with a total of 1563 annotated genes. Selected top 9 GO class for the mapping display of molecular functions, cellular components, and biological processes (Figures 2(a)–2(c)).

**Figure 3:**
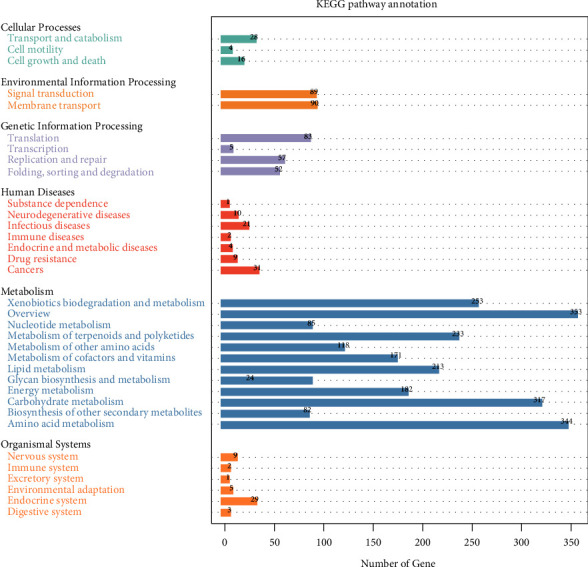
HJW gene KEGG enrichment bar graph. The abscissa represents the number of genes, and the ordinate represents the code of each functional class of level 1 in the database. For the explanation of the code, see the corresponding legend.

KEGG enrichment analysis showed that differentially expressed genes were significantly enriched in 6 signaling pathways, including cellular processes, environmental information processing, genetic information processing, human diseases, metabolism, and organismal systems (Supplementary Table 5). The KEGG enrichment analysis bar graph (Figure 3) has significantly enriched the abovementioned six categories of secondary classification entries, including 34 aspects such as transport and catabolism, replication and repair, immune diseases, cancers, and immune system.

**Figure 4:**
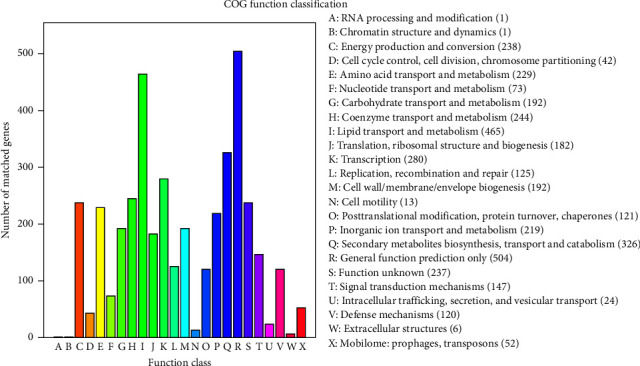
HJW gene COG function enrichment histogram. The abscissa is the COG function classification, and the ordinate is the number of matched genes.

The predicted amino acid sequences of the HJW genome were compared with those in the COG database, and HJW genes and corresponding functional annotation information were combined to obtain the annotation results (Supplementary Table 6). Since there could be more than one sequence alignment result for each sequence, to ensure its biological significance, an optimal alignment result is retained as the annotation of the gene when annotating (Figure 4).

**Figure 5:**
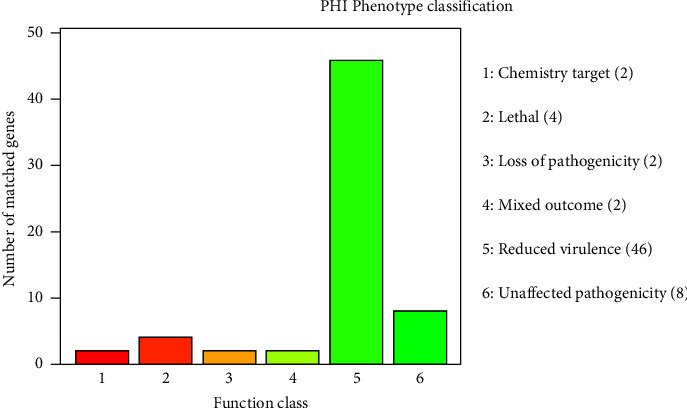
HJW gene PHI function enrichment histogram. The abscissa is the COG function classification, and the ordinate is the number of matched genes.

In addition, the PHI phenotypic mutation types of HJW were counted through the pathogen and host interaction database (PHI) annotations, and 46 genes related to virulence decline and 2 genes related to virulence loss were found (Figure 5). Several PHI phenotypic classifications such as lethal and loss of pathogenicity were also annotated (Supplementary Table 7). Based on the annotation results of the protein sequence function database, the secretory system-related proteins were extracted for annotation, and 9 T7SS (type 7 secretion systems) effector proteins were predicted in the HJW genome.

### 3.6. Results of Phylogenetic Analysis

**Figure 6:**
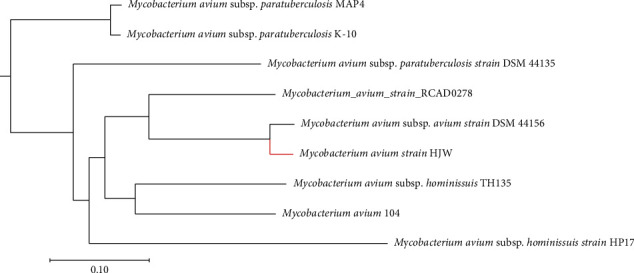
Phylogenetic analysis of nine complete genomic sequences.

The result of the constructed phylogenetic tree is shown in Figure 6. According to the result, the subspecies distribution of *Mycobacterium avium* can be seen. The HJW strain used for genome sequencing in this part of the experiment is in the same branch as *Mycobacterium avium* subsp. *avium* strain DSM 44156 and is closely related to the MAA subspecies. In addition, as a *Mycobacterium avium* subsp. *avium* strain isolated from China, RCAD0278 belongs to the same branch as HJW strain on the phylogenetic tree [53]. Insertion sequences IS1311 and IS901 were found in both strains. The IS1311 repeat number and sequence of the two are completely consistent. The number of repetitions in IS901 was different, 16 in HJW, and 15 in RCAD0278. In addition, there was a nucleotide polymorphism at 128 of the coding region of HSP65 gene in both of them, which was G in RCAD0278 and T in HJW.

### 3.7. Results of the Virulence Factors Analysis

**Figure 7:**
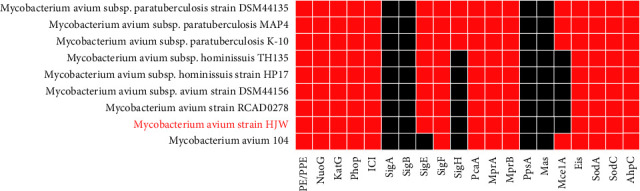
Distribution of virulence genes from different *Mycobacterium* species.

The analysis results of the distribution of the virulence factors in each subspecies of *Mycobacterium avium* are shown in Figure 7. Although there are individual differences in the genes carried by each subspecies, there is no obvious rule. Among the identified HJW strains, major virulence factors such as PE/PPE, *SigE*, *SigF*, and *Phop* were detected, further indicating the potential pathogenicity of the HJW strain.

## 4. Discussion

Although the four subspecies of *Mycobacterium avium* are closely related, they still represent distinct organisms and exhibit phenotypic diversity. The pathogenicity and host preference of each subspecies are different, ranging from environmental bacteria that can cause opportunistic infections in immunocompromised individuals to birds and other domestic and wild pathogens of animals [54–58]. Unlike other *Mycobacterium avium* subspecies, the host of MAA infection is mainly birds, especially domestic poultry, which are highly sensitive to MAA and are usually the first to contract the disease and work as the main reservoir of the bacteria. Infected birds can spread MAA to susceptible animals via feces, wild birds, rodents, or through fecal polluted environment [59]. In addition to birds, MAA can also infect various mammals, including small rodents. People with low immunity are also in the scope of infection. In pigs and cattle, the most common sites of infection with MAA are mostly in the head and mesenteric lymph nodes, and occasionally, there are a few lesions in the inguinal lymph nodes of pigs [30].

This subspecies has high virulence potential. Christine Goepfert et al. report on four veal calves suffering from an unusual form of intestinal mycobacterios that was due to infection with MAA. Clinical signs were symptoms of otitis media, fever, and chronic weight loss. Pathological damage was similar to the symptoms caused by paratuberculosis, with segmental thickening in ileum, cecum, and proximal colon, accompanied by multifocal ulcer caused by granulomatous inflammation [60]. In an early study by the Iranian Veterinary Organization (IVO) showed that MAA was frequently isolated from lymph nodes of cattle that were slaughtered following a positive result in the routine tuberculin test [29]. In addition, the MAA is able to infect cattle and develop hypersensitivity towards bovine tuberculin in the skin test, to form tuberculous lesions. Ultimately affect the interpretation of the results. However, after MAA infection in horses, the main manifestations were found in various lymph nodes [32]. As mentioned above, MAA can cause diseases in animals such as pigs and cattle and cause serious economic losses. Therefore, it is very important to diagnose this kind of infection correctly and quickly.

Currently, although few MAA infections have been reported in human clinical cases, this may be related to host preference. In the last few years, more attention has been paid to MAC study in China, and most of the reports have focused on MAP research [20, 21, 24]. The aim of this study was to isolate MAP from fecal samples of diarrheal lean cattle suspected of paratuberculosis, but instead a strain of MAA was isolated. This result suggests that cattle can also be infected with MAA. At the same time, it was suggested that the infected cattle may have been doubly infected with MAP and MAA. However, because only MAA was isolated, it is speculated that MAP has not yet shed from intestinal mucosa. Of course, it is a pity that this study lacks follow-up research on physiological state and intestinal pathological changes of sick cattle.

Genetic analysis assay is a valuable method for differentiating the MAC subspecies. However, differentiation between the MAC subspecies is not possible through the 16S rRNA sequence. Furthermore, although amplification of the insertion sequences IS1311, IS901, IS900, and DT1 gene can discriminate the MAP and MAH subspecies, but it cannot distinguish the MAA and MAS [61]. In this paper, for definitive identification of the strain as MAA subspecies, Gram staining, Ziehl–Neelsen acid-resistant staining, and insertion sequence amplification were used, and the results were further clarified with whole genome sequencing and evolutionary tree analysis.

In addition, through a series of software combined with database analysis, we have predicted the HJW genome composition and function, including repetitive sequence analysis. Repetitive sequences are a component of gene regulation network and play a pivotal role in the growth and metabolism of species, genetic evolution and mutation, gene island prediction, gene island pathogenic mechanism and organism adaptability, etc. Biological functions are interrelated and these analyses can provide a certain basis for subsequent related in-depth research. In addition, a total of 9 effector proteins of type VII secretion system were predicted in the HJW genome. This secretion system is indispensable for the virulence and survival of MAA [62, 63]. Pathogenic bacteria can multiply and diffuse in the host by secreting virulence factors and other macromolecular substances. Earliest research on the bacterial secretion system mainly focused on Gram-negative bacteria, and it was found that the type I–VI secretion systems were involved for pathogenicity. With the deepening of the research, type VII has been found to be important in Gram-positive bacteria for pathogenicity [64]. Secretion system (type VII secretion system, T7SS), T7SS was first discovered in mycobacteria. One of the main functions of T7SS is to secrete PE/PPE family proteins. The PE/PPE protein family is unique to mycobacteria. Relevant studies have shown that members of this family play a role in host-pathogen interaction and pathogenesis [65, 66]. Therefore, we have tabulated the distribution of PE/PPE family among *Mycobacterium avium* subspecies for comparison and analysis (Figure 7).

The results of phylogenetic analysis show that the HJW strain had at least 89% sequence coverage to the other *Mycobacterium avium* subspecies strains and had the highest sequence similarity with *Mycobacterium avium* subsp. *avium* strain DSM 44156. *Mycobacterium avium* subsp. *avium* strain DSM 44156 is a standard reference strain for MAA, originally a serotype 2 strain isolated from the liver of diseased hens [67]. Phylogenetic analysis also revealed that the isolate clustered with *Mycobacterium avium* subsp. *avium* DSM 44156. In addition, HJW was closely related to RCAD 0278 and belonged to the same branch. RCAD0278 was isolated from a case of duck tuberculosis in Sichuan, China. According to the genomic sequence information, the insertion sequences IS1311 and IS901 were both carried by this strain and HJW strain. Although the two strains were isolated from China, their geographical locations were far apart and their hosts were different, which indicated that MAA was prevalent in China. These results should remind researchers in China to pay more attention to the prevalence of MAA. Nevertheless, further investigations of MAA are necessary to improve interpretation of the pathogenicity of this strain and enrich the genome information of this pathogen.

Comparative studies on the genomes of different mycobacteria show that the gene products that make up the cell wall and gene families encoding acidic glycine-rich PE/PPE proteins are the main reasons for the differences between species [68]. PE and PPE mainly exist in pathogenic mycobacterium, and the regulation of host innate immunity is a potential component of antituberculosis vaccine, which is related to the pathogenesis of mycobacterium. The PPE and PE gene families account for 10% of the *Mycobacterium tuberculosis* genome, encode a great variety of proteins of unknown function, and are a major source of differences between the *Mycobacterium tuberculosis* and *Mycobacterium bovis* genomes [69]. The PE/PPE gene family may play an important role in host-pathogen interactions, granuloma persistence, and generation of antigenic variation and immune evasion [70–72]. But, their role in mycobacteria is mostly unknown. At present, some studies have shown that members of PE and PPE families are related to virulence [73].

Sigma factors are a family of regulatory proteins that are a critical part of the immunopathology of *Mycobacterium tuberculosis*. They are mainly responsible for promoter recognition and mediate the differential expression of various genes including virulence factors. Sigma factors are important for the rapid adaptation of *Mycobacterium tuberculosis* to the changes in the surrounding environment. The more kinds of Sigma factor encoded, the stronger the ability of *Mycobacterium tuberculosis* to adapt to the changes in the surrounding environment [74]. *Mycobacterium tuberculosis* encodes 13 Sigma factors named Sigma A–M. The main factors related to persistent *Mycobacterium tuberculosis* infection are *SigA*, *SigF*, and *SigH*. The *Phop* gene is a key gene transcribed in host cells by various intracellular pathogenic bacteria. As a response regulator capable of regulating biosynthesis and gene expression, it has multiple functions in the virulence and persistence of *Mycobacterium tuberculosis* and is critical for virulence. In the comparative genomic analysis, a variety of other important and common virulence genes were also compared. Shortly, the virulence factors of these strains of mycobacteria are not exactly the same, and there are slight differences. Subtle differences such as the existence or deletion of different virulence factors may be the reasons for the pathogenesis differences among mycobacterium species; so, these substantial differences are also helpful to explain the important phenotypic differences among MAC members.

## 5. Conclusions

In summary, we first identified a strain of MAA from bovine in China. In addition, we successfully produced a circular chromosome of the MAA strain HJW. The genome was closely related to *Mycobacterium avium* subsp. *avium* strain DSM 44156. The present work contributes to the understanding of the epidemiology of MAA in China and the results of complete genomic sequencing may also help to classify the subspecies of *Mycobacterium avium*.

## Data Availability

The data that support the findings of this study are available in GenBank at NCBI, reference number [CP028731].

## Supplementary Materials

Supplementary Materials Supplementary Figure 1: (A) Agarose gel electrophoresis of PCR products amplified using primers 16S rRNA gene. Lane (*M*) 2000 bp molecular size marker; lane 1: HJW; lane 2: *M. avium* subsp. *paratuberculosis*s (MAP-10) (B) Agarose gel electrophoresis of PCR products amplified using primers IS1311 rRNA gene. Lane (*M*) 2000 bp molecular size marker; lane 1: HJW; lane 2: *M. avium* subsp. *paratuberculosis* (MAP-10). (C) Agarose gel electrophoresis of PCR products amplified using primers IS901 rRNA gene. Lane (*M*) 2000 bp molecular size marker; lane 1: HJW. (D) Agarose gel electrophoresis of PCR products amplified using primers DT1. Lane (*M*) 2000 bp molecular size marker; lane 1: HJW. (E) Agarose gel electrophoresis of PCR products amplified using primers IS900 gene. Lane (*M*) 2000 bp molecular size marker; lane 1: *M. avium* subsp. *paratuberculosis* (MAP-10). Supplementary Table 1: the result of interspersed nucleotide repeated sequences. Supplementary Table 2: the result of tandem nucleotide repeated sequences. Supplementary Table 3: the result of Genomics island. Supplementary Table 4: HJW GO Enrichment Analysis Results Table. Supplementary Table 5: KEGG Enrichment Analysis Results Table. Supplementary Table 6: COG Enrichment Analysis Results Table. Supplementary Table 7: PHI Enrichment Analysis Results Table.
