# Supplementary material for: Identification and Phylogenetic Analysis of Mycobacterium avium subsp. avium Strain Isolated from Cow
Source: Transbound Emerg Dis. 2023 Jun 1;2023:5384079. doi: 10.1155/2023/5384079 (PMC12017052; doi:10.1155/2023/5384079)
Supplement: Supplementary Materials — Supplementary Figure 1: (A) Agarose gel electrophoresis of PCR products amplified using primers 16S rRNA gene. Lane (M) 2000 bp molecular size marker; lane 1: HJW; lane 2: M. avium subsp. paratuberculosiss (MAP-10) (B) Agarose gel electrophoresis of PCR products amplified using primers IS1311 rRNA gene. Lane (M) 2000 bp molecular size marker; lane 1: HJW; lane 2: M. avium subsp. paratuberculosis (MAP-10). (C) Agarose gel electrophoresis of PCR products amplified using primers IS901 rRNA gene. Lane (M) 2000 bp molecular size marker; lane 1: HJW. (D) Agarose gel electrophoresis of PCR products amplified using primers DT1. Lane (M) 2000 bp molecular size marker; lane 1: HJW. (E) Agarose gel electrophoresis of PCR products amplified using primers IS900 gene. Lane (M) 2000 bp molecular size marker; lane 1: M. avium subsp. paratuberculosis (MAP-10). Supplementary Table 1: the result of interspersed nucleotide repeated sequences. Supplementary Table 2: the result of tandem nucleotide repeated sequences. Supplementary Table 3: the result of Genomics island. Supplementary Table 4: HJW GO Enrichment Analysis Results Table. Supplementary Table 5: KEGG Enrichment Analysis Results Table. Supplementary Table 6: COG Enrichment Analysis Results Table. Supplementary Table 7: PHI Enrichment Analysis Results Table. [file 5384079.f1.zip › Supplementary Table 1. The result of interspersed nucleotide repeated sequences.pdf]

Type	Number	Total Length (bp)	In Genome(%)	Average length (bp)
LTR	16	1200	0.0242	75
DNA	5	477	0.0096	95
LINE	2	274	0.0055	137
SINE	7	352	0.0071	50
Total	30	2251	0.0454	77
