# Supplementary material for: Identification and Phylogenetic Analysis of Mycobacterium avium subsp. avium Strain Isolated from Cow
Source: Transbound Emerg Dis. 2023 Jun 1;2023:5384079. doi: 10.1155/2023/5384079 (PMC12017052; doi:10.1155/2023/5384079)
Supplement: Supplementary Materials — Supplementary Figure 1: (A) Agarose gel electrophoresis of PCR products amplified using primers 16S rRNA gene. Lane (M) 2000 bp molecular size marker; lane 1: HJW; lane 2: M. avium subsp. paratuberculosiss (MAP-10) (B) Agarose gel electrophoresis of PCR products amplified using primers IS1311 rRNA gene. Lane (M) 2000 bp molecular size marker; lane 1: HJW; lane 2: M. avium subsp. paratuberculosis (MAP-10). (C) Agarose gel electrophoresis of PCR products amplified using primers IS901 rRNA gene. Lane (M) 2000 bp molecular size marker; lane 1: HJW. (D) Agarose gel electrophoresis of PCR products amplified using primers DT1. Lane (M) 2000 bp molecular size marker; lane 1: HJW. (E) Agarose gel electrophoresis of PCR products amplified using primers IS900 gene. Lane (M) 2000 bp molecular size marker; lane 1: M. avium subsp. paratuberculosis (MAP-10). Supplementary Table 1: the result of interspersed nucleotide repeated sequences. Supplementary Table 2: the result of tandem nucleotide repeated sequences. Supplementary Table 3: the result of Genomics island. Supplementary Table 4: HJW GO Enrichment Analysis Results Table. Supplementary Table 5: KEGG Enrichment Analysis Results Table. Supplementary Table 6: COG Enrichment Analysis Results Table. Supplementary Table 7: PHI Enrichment Analysis Results Table. [file 5384079.f1.zip › Supplementary Table 3. The result of Genomics island.pdf]

| GIs_id | Sequence_id | Sequence_start | Sequence_end | GIs_length |
|---|---|---|---|---|
| GIs001 | Chr1 | 380,225 | 394,394 | 14,170 |
| GIs002 | Chr1 | 699,965 | 706,696 | 6,732 |
| GIs003 | Chr1 | 994,657 | 1,002,486 | 7,830 |
| GIs004 | Chr1 | 1,192,027 | 1,208,479 | 16,453 |
| GIs005 | Chr1 | 1,604,230 | 1,611,947 | 7,718 |
| GIs006 | Chr1 | 1,820,178 | 1,827,769 | 7,592 |
| GIs007 | Chr1 | 2,198,602 | 2,210,980 | 12,379 |
| GIs008 | Chr1 | 2,215,970 | 2,227,883 | 11,914 |
| GIs009 | Chr1 | 3,271,901 | 3,284,107 | 12,207 |
| GIs010 | Chr1 | 3,680,040 | 3,708,996 | 28,957 |
| GIs011 | Chr1 | 3,906,462 | 3,916,677 | 10,216 |
| GIs012 | Chr1 | 4,164,777 | 4,169,593 | 4,817 |
| GIs013 | Chr1 | 4,193,317 | 4,208,751 | 15,435 |
